# Association Between Serum Gamma-Glutamyl Transferase Levels and Angiographic Severity of Coronary Artery Disease in Patients With Chronic Coronary Syndrome

**DOI:** 10.7759/cureus.97535

**Published:** 2025-11-23

**Authors:** A K M Imtiaz Akand, Iffat Ara Jurfa, Md Fazlur Rahman, Ahsan Habib, Chetan Upadhyaya

**Affiliations:** 1 Department of Cardiology, Bangladesh Medical University, Dhaka, BGD; 2 Department of Cardiology, North Wales Cardiac Centre, Bodelwyddan, GBR; 3 Department of Internal Medicine, Bangladesh Medical University, Dhaka, BGD

**Keywords:** chronic coronary syndrome, coronary artery disease, gamma-glutamyl transferase, gensini score, oxidative stress

## Abstract

Background: Chronic coronary syndrome (CCS) is a common clinical manifestation of coronary artery disease (CAD) resulting from an imbalance between myocardial oxygen supply and demand, most often due to atherosclerotic obstruction. Serum gamma-glutamyl transferase (GGT), a biomarker of oxidative stress, has been implicated in the pathogenesis of atherosclerosis and may correlate with the severity of CAD.

Objective: To evaluate the relationship between serum GGT levels and the angiographic severity of CAD in patients with CCS.

Methods: This cross-sectional observational study was conducted in the Department of Cardiology, Bangladesh Medical University (BMU), formerly known as Bangabandhu Sheikh Mujib Medical University, Dhaka, Bangladesh, from October 2019 to September 2020. A total of 50 patients with CCS who underwent coronary angiography were included. Participants were divided into two groups: Group A (25 patients with severe CAD, Gensini score ≥36) and Group B (25 patients with normal coronary arteries, Gensini score <36). Serum GGT levels were measured in the Department of Biochemistry, and angiographic severity was assessed using the Gensini scoring system. Statistical analyses were performed using Statistical Product and Service Solutions (SPSS, version 23.0; IBM SPSS Statistics for Windows, Armonk, NY).

Results: The mean serum GGT level was significantly higher in Group A (64.28 ± 14.36 U/L) than in Group B (32.80 ± 5.04 U/L) (p = 0.001). A strong positive correlation was found between GGT level and both the number of affected vessels (r = 0.758, p = 0.001) and the Gensini score (r = 0.923, p = 0.001), indicating that higher GGT levels were associated with greater angiographic severity of CAD.

Conclusion: Serum GGT level showed a significant positive correlation with the angiographic severity of CAD in patients with CCS, suggesting its potential role as a biomarker for disease severity and risk stratification.

## Introduction

Coronary artery disease (CAD) remains one of the leading causes of morbidity and mortality worldwide. In Asian populations, CAD tends to occur at a younger age, with greater severity compared to Western populations. This epidemiological difference highlights the need to identify additional biomarkers that could help explain this higher risk and improve early diagnosis and risk stratification [1]. Despite extensive research, only a limited number of biomarkers have been validated for the early detection of CAD, emphasizing the importance of exploring novel indicators.

Cardiovascular disease (CVD) encompasses a range of disorders affecting the heart and blood vessels, including chronic coronary syndrome (CCS) [2]. The prevalence of CAD in Bangladesh, including rural populations, is comparable to that seen in developed countries. Male sex, higher socioeconomic status, hypertension, and diabetes mellitus have been identified as independent risk factors [3].

Gamma-glutamyl transferase (GGT) has traditionally been considered a biomarker of hepatobiliary disease and alcohol consumption. However, GGT is also produced by extrahepatic tissues, such as the kidneys, lungs, lymphocytes, fibroblasts, and epididymis [4]. Experimental studies suggest that GGT plays a role in extracellular glutathione metabolism, a major antioxidant system in humans, and elevated GGT levels may reflect increased oxidative stress, a key factor in atherosclerosis [5].

Serum GGT levels have been shown to correlate with cardiovascular risk factors, including obesity, hypertension, type 2 diabetes, and dyslipidemia [6]. These findings suggest that GGT may serve as a novel biomarker for CVD. Evaluating serum GGT levels in patients with CCS undergoing angiography may therefore help assess disease severity and potentially improve early diagnosis. However, limited data from Bangladesh exist regarding the relationship between GGT and angiographic CAD severity, highlighting an important research gap.

This study aimed to determine the correlation between serum GGT levels and the angiographic severity of CAD, as assessed by the Gensini score, in patients with CCS.

## Materials and methods

Study design and population

This cross-sectional observational study was conducted in the Department of Cardiology, Bangladesh Medical University (BMU), Dhaka, Bangladesh, from October 2019 to September 2020. The study protocol was approved by the Institutional Review Board (IRB) of BMU. Informed written consent was obtained from all participants after a full explanation of the study’s purpose and procedures.

A total of 50 patients with a diagnosis of CCS who underwent coronary angiography were prospectively enrolled through consecutive sampling. This study included adults ≥18 years with CCS who underwent elective coronary angiography. Exclusion criteria involved liver disease (alanine transaminase (ALT) or aspartate aminotransferase (AST) >2× upper limit), alcohol intake: >14 drinks/week (men), >7 drinks/week (women), medications affecting GGT (e.g., antiepileptics), acute infection, or pregnancy. Based on angiographic findings, participants were stratified into two groups: Group A, consisting of patients with severe coronary artery disease (Gensini score ≥ 36), and Group B, consisting of patients with angiographically normal coronary arteries (Gensini score < 36).

Data collection

Comprehensive demographic data (age, sex, BMI), cardiovascular risk factors (smoking, hypertension, diabetes, dyslipidemia, family history of CAD, and prior coronary events or revascularization), and investigation results (12-lead ECG, serum creatinine, and echocardiography findings) were systematically recorded. These details were obtained using a structured proforma to ensure consistency and completeness of data collection.

Fasting serum GGT levels were measured via the automated IFCC enzymatic method. The reference range for normal GGT was defined as <55 IU/L for men and <38 IU/L for women. LVEF was measured by Simpson’s biplane method. Coronary angiography was performed by experienced interventional cardiologists according to standard clinical practice guidelines. 

Assessment of angiographic severity

The severity of coronary artery disease was evaluated using the Gensini score, which quantifies the degree of luminal narrowing and the importance of lesion location in the coronary arterial tree. Gensini scoring was done by two independent cardiologists. A score <36 was considered non-severe CAD, while ≥36 indicated severe CAD [7]. The correlation between serum GGT levels and Gensini score was analyzed in both groups.

Statistical analysis

Data analysis was performed using Statistical Product and Service Solutions (SPSS, version 23.0; IBM SPSS Statistics for Windows, Armonk, NY). Normality was assessed using Shapiro‑Wilk testing. Parametric or non‑parametric tests were applied accordingly. Continuous variables were expressed as mean ± standard deviation (SD), and categorical variables were expressed as frequencies and percentages. The chi-square test was used to compare categorical variables, while the unpaired t-test assessed differences in means between groups. The Pearson correlation coefficient was applied to evaluate the relationship between serum GGT levels and the Gensini score. The Spearman correlation coefficient was used to assess the association between GGT levels and the number of affected vessels. Exact p‑values are reported, and a p-value < 0.05 was considered statistically significant. Receiver operating characteristic (ROC) analysis was performed to assess the diagnostic performance.

## Results

A total of 50 patients with CCS were included in this study. They were divided equally into two groups: Group A (severe coronary artery disease, n = 25) and Group B (normal coronary arteries, n = 25).

Demographic and clinical characteristics

Both the study groups were similar in age, sex, and BMI distribution (Table 1).

**Table 1:** Demographic characteristics of the study population ns=not significant. P-value: p<0.05 (threshold for statistical significance). Age → Unpaired t-test; BMI → Unpaired t-test; Sex → Chi-square test

Variables	Group A (n=25)		Group B (n=25)		P-value
	n	%	n	%	
Age in years (Mean ± SD)	53.77±8.75		55.60±6.61		0.408^ns^
Sex					
Male	14	56	17	68	0.41
Female	11	44	08	32	
BMI (kg/m^2^)	28.57±2.53		28.00±2.12		0.392^ns^

Presenting symptoms

All patients in both groups presented with chest pain. Shortness of breath was observed in nine (36.0%) patients in Group A and five (20.0%) in Group B (p = 0.207). Palpitations were reported in nine (36.0%) patients in Group A and eight (32.0%) in Group B (p = 0.765). Fatigue was present in five (20.0%) patients in Group A and four (16.0%) in Group B, showing no significant difference (p > 0.05) (Table 2).

**Table 2:** Distribution of the study patients by symptoms (n=50) ns=not significant. P-value: p< 0.05 ( threshold for statistical significance). All variables in Table 2 - chi-square test

Symptoms	Group A (n=25)		Group B (n=25)		P-value
	n	%	n	%	
Chest pain	25	100	25	100	_
Shortness of breath	9	36	5	20	0.207 (ns)
Palpitation	9	36	8	32	0.765 (ns)
Fatigue	5	20	4	16	0.500 (ns)

Risk factor profile

Hypertension was present in 20 (80.0%) patients in Group A and 16 (64.0%) in Group B (p > 0.05). Dyslipidemia (an elevation of plasma cholesterol, triglycerides (TGs), or both, or a low high-density lipoprotein cholesterol (HDL-C) level that contributes to the development of atherosclerosis) was significantly more common in Group A (80.0%) compared to Group B (48.0%) (p = 0.018). Obesity(chronic complex disease defined by excessive fat deposits that can impair health) and smoking status did not differ significantly between the groups (p > 0.05) (Table 3).

**Table 3:** Distribution of the study patients by risk factors (n = 50) s=significant; ns=not significant. P-value: p < 0.05 (threshold for statistical significance). All variables in Table 3 - chi-square test

Risk factors	Group A (n=25)		Group B (n=25)		P-value
	N	%	n	%	
Obesity	11	44.0	7	28.0	0.238^ns^
Hypertension	20	80.0	16	64.0	0.207^ns^
Dyslipidemia	20	80.0	12	48.0	0.018^s^
Smoking	12	48.0	14	56.0	0.571^ns^

Left ventricular ejection fraction (LVEF)

LVEF was measured using Simpson's biplane method and was blinded to angiographic findings to minimize bias. The mean LVEF was significantly lower in Group A compared to Group B (49.85 ± 7.46% vs. 54.72 ± 5.81%; p = 0.013) (Table 4).

**Table 4:** Distribution of the study patients by left ventricular ejection fraction (LVEF) (n = 50) s=significant. P-value: p < 0.05 (threshold for statistical significance) reached from the unpaired t-test. LVEF categories → chi-square test; Mean LVEF (mean ± SD) → Unpaired t-test

LVEF (%)	Group A (n=25)		Group B (n=25)		P-value
	n	%	n	%	
30-39	2	8.0	0	0.0	
40-49	11	44.0	5	20.0	
50-70	12	48.0	20	80.0	
Mean±SD	49.85±7.46		54.72±5.81		0.013^s^

Serum GGT levels

The mean serum GGT level was markedly higher in Group A (64.28 ± 14.36 U/L) compared to Group B (32.80 ± 5.04 U/L), a difference that was statistically significant (p = 0.001). Abnormal GGT levels (≥55 U/L for men and ≥38 U/L for women) were observed in 20 (80.0%) patients in Group A, compared to only one (4.0%) patient in Group B (p = 0.001) (Table 5).

**Table 5:** Distribution of the study patients by gamma-glutamyl transferase (GGT) (n = male <55; female <38) s=significant. P-value: p <0.05 (threshold for statistical significance) reached from the unpaired t-test. Normal (male <55 U/L and female <38 U/L), abnormal (male ≥55 U/L and female ≥38 U/L) GGT category (normal/abnormal) → Chi-square test. Mean GGT (mean ± SD) → Unpaired t-test

GGT	Group A (n=25)		Group B (n=25)		P-value
	n	%	n	%	
Normal	5	20.0	24	96.0	
Abnormal	20	80.0	1	4.0	
Mean±SD	64.28±14.36		32.80±5.04		0.001^s^

Coronary angiographic findings

**Figure 1:**
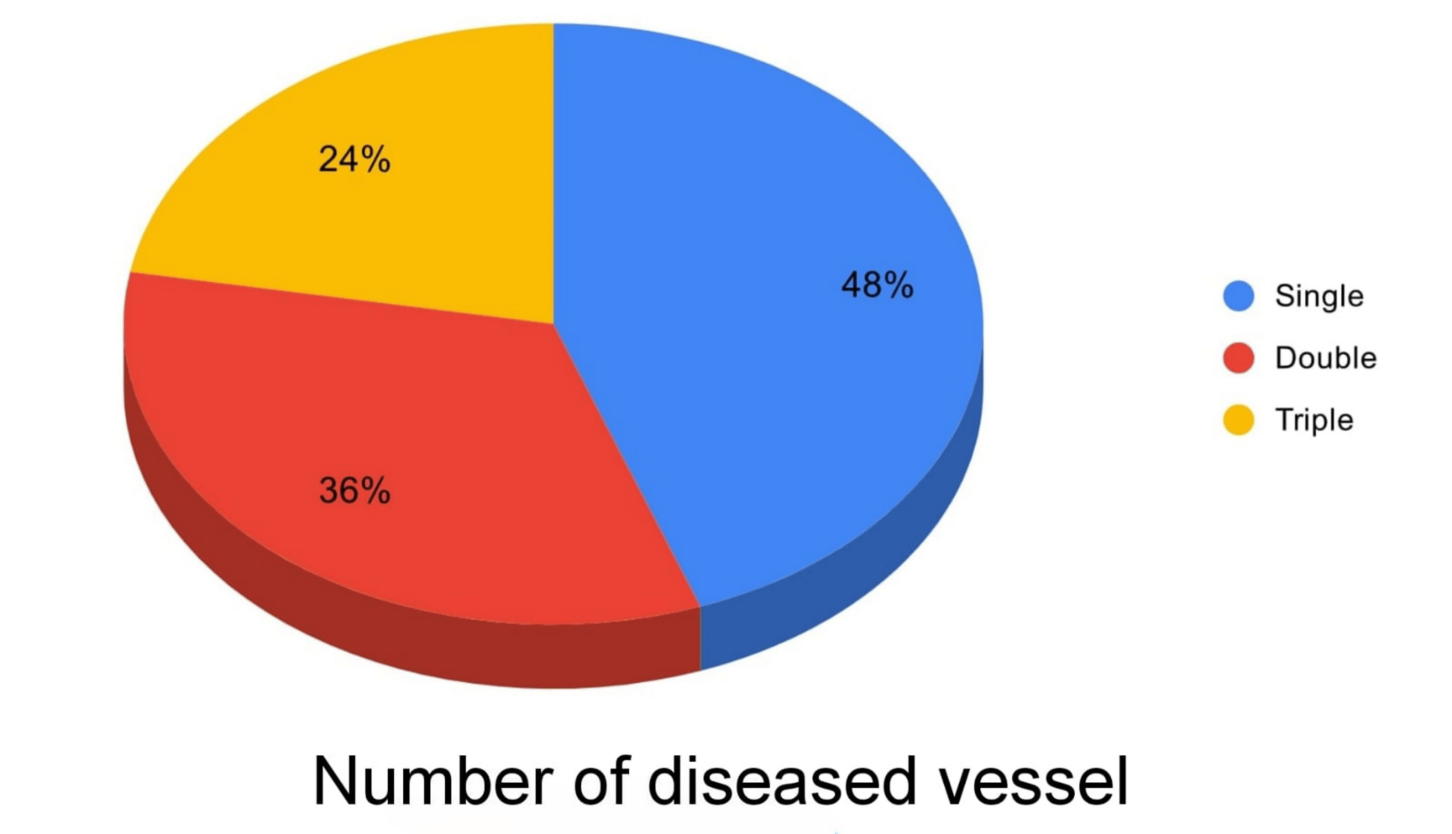
Pie chart showing the percentage of the number of affected vessels of the patients with severe coronary artery disease (n = 25)

**Figure 2:**
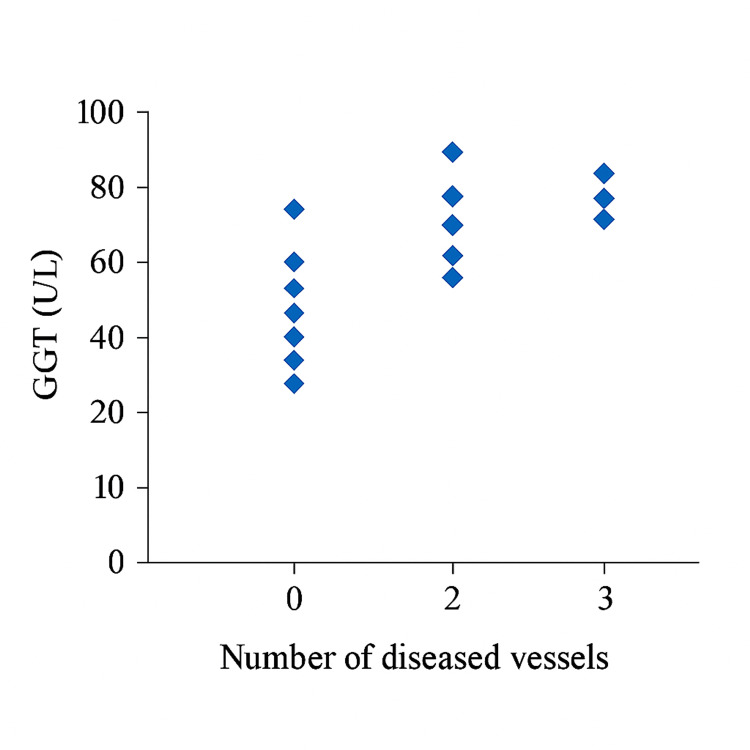
Scatter diagram showing a positive significant correlation (r = 0.758; p = 0.001) between gamma-glutamyl transferase (GGT) and the number of affected vessels in patients with severe coronary artery disease Pearson’s correlation coefficient assessed the relationship between GGT levels and the Gensini score. Spearman’s correlation coefficient evaluated the association between GGT levels and the number of affected vessels. A p-value <0.05 was considered statistically significant.

Among patients with severe CAD (Group A), 12 (48.0%) had single-vessel disease, nine (36.0%) had double-vessel disease, and four (16.0%) had triple-vessel disease (Figure 1). A significant positive correlation was found between serum GGT levels and the number of affected vessels (r = 0.758; p = 0.001; Figure 2). Additionally, there was a strong positive correlation between serum GGT levels and Gensini score (r = 0.923; p = 0.001).

ROC analysis

The diagnostic performance of serum GGT for identifying severe CAD was evaluated using ROC analysis. The area under the curve (AUC) was 0.94, indicating excellent accuracy in distinguishing severe from non-severe CAD.

## Discussion

The primary finding of this study is that serum GGT levels are significantly associated with angiographic CAD severity in CCS patients. The mean serum GGT level was significantly elevated in Group A (64.28 ± 14.36 U/L) compared to Group B (32.80 ± 5.04 U/L) (p = 0.001). This suggests that higher serum GGT levels are associated with increased severity of coronary artery disease. Arasteh et al. similarly demonstrated significantly elevated GGT levels among patients with angiographically confirmed CAD compared to controls [8]. Elevated GGT may reflect oxidative stress and inflammation within the vascular endothelium, contributing to atherosclerotic plaque formation and progression.

Among patients with severe CAD, 48.0% had single-vessel disease, 36.0% had double-vessel disease, and 16.0% had triple-vessel disease, findings comparable to those of Taimur et al. [9]. In our study, serum GGT levels showed a strong positive correlation with both the number of affected vessels (r = 0.758; p = 0.001) and the Gensini score (r = 0.923; p = 0.001), indicating that GGT may serve as a marker of disease burden. While Demircan et al. [10] reported no significant relationship between GGT levels and the number of affected vessels, Duran et al. [11] observed a positive correlation (r = 0.355; p < 0.001), supporting the findings of the present study. The observed discrepancies across studies may be attributed to differences in sample size, population characteristics, and methods used to quantify angiographic severity.

In this study, the mean age of patients in the severe CAD group (Group A) was 53.77 ± 8.75 years, while in the normal coronary group (Group B), it was 55.60 ± 6.61 years. The difference was not statistically significant (p > 0.05), suggesting that age was not a confounding factor in the association between serum GGT levels and angiographic severity. Arasteh et al. reported comparable findings, with mean ages of 51.4 ± 14.5 years in the CAD group and 54.5 ± 6.6 years in the healthy control group [8]. Similarly, Huang et al. found no significant age difference between patients with acute coronary syndrome (ACS) and controls [12].

Male predominance was noted in both groups (56.0% in Group A and 68.0% in Group B), consistent with previous reports that men are more frequently affected by CAD [7]. The mean body mass index (BMI) did not differ significantly between the two groups (28.57 ± 2.53 kg/m² vs. 28.00 ± 2.12 kg/m²; p > 0.05). These findings align with those of Arasteh et al. [7] and Huang et al. [8], who observed similar BMI values among CAD and control participants, suggesting that BMI alone may not predict angiographic disease severity.

Hypertension and dyslipidemia were the most prevalent cardiovascular risk factors in this study. Hypertension was present in 80.0% of patients in Group A and 64.0% in Group B (p > 0.05), while dyslipidemia was significantly higher among patients with severe CAD (80.0%) compared to those with normal coronary arteries (48.0%) (p < 0.05). These results are in agreement with Huang et al. [8], who found that dyslipidemia was more prevalent in patients with ACS, emphasizing the role of lipid abnormalities in coronary atherosclerosis.

LVEF was significantly lower in patients with severe CAD (49.85 ± 7.46%) compared to those with normal coronary arteries (54.72 ± 5.81%; p = 0.013). This finding corresponds with that of Demircan et al. [9], who reported reduced LVEF among patients with extensive coronary artery involvement, reflecting impaired myocardial function due to ischemic damage.

The strong correlation between GGT and angiographic indices of CAD severity highlights the enzyme’s potential role in cardiovascular risk assessment. GGT participates in glutathione metabolism and is closely linked to oxidative stress, a key mechanism in endothelial dysfunction and plaque instability. These findings suggest that elevated serum GGT levels may not only reflect hepatic or metabolic disturbances but also indicate increased oxidative stress and subclinical vascular inflammation associated with CAD progression.

Limitations

The main limitations of this study include its relatively small sample size in a single centre and cross-sectional design, which preclude causal inferences. Additional limitations include a lack of multivariate adjustment due to limited sample size and possible interobserver variability in Gensini scoring. Potential confounders such as alcohol consumption and undiagnosed hepatic dysfunction, although addressed in the exclusion criteria, could influence GGT levels. Further future prospective studies with larger and more diverse populations are recommended to validate these findings and explore the prognostic value of GGT in CAD.

## Conclusions

This study demonstrated a significant positive correlation between serum GGT levels and the angiographic severity of CAD in patients with CCS. Serum GGT levels were markedly elevated among patients with chronic stable angina who exhibited severe CAD, suggesting that GGT may serve as a useful biomarker for assessing disease severity. Further longitudinal follow-up is needed to determine prognostic implications of elevated GGT for adverse cardiac outcomes, and large-scale, multicenter studies are warranted to validate these findings and to establish the prognostic value of GGT as a novel, cost-effective marker in CAD.

## References

[1] Gültürk İ, Sönmezöz GB, Aksoy S, Polat H (2024). Comparison of coronary artery calcium score and serum calcium, phosphorus, and gamma-glutamyl transferase levels in patients with coronary artery imaging by multi-sectional computed tomography with chronic ischemic heart disease prediagnosis. Istanbul Med J.

[2] Singh KK, Kapoor A, Khanna R (2022). Serum gamma-glutamyltransferase (GGT) in coronary artery disease: exploring the Asian Indian connection. Ann Card Anaesth.

[3] Sanchis-Gomar F, Perez-Quilis C, Leischik R, Lucia A (2016). Epidemiology of coronary heart disease and acute coronary syndrome. Ann Transl Med.

[4] Banerjeel SK, Ahmed CM, Rhaman MM, Chowdhury MM, Sayeed MA (2017). Coronary artery disease in a rural population of Bangladesh: is dyslipidemia or adiposity a significant risk?. IMC J Med Sci.

[5] Xuan C, Li J, Liu RH (2023). Association between serum gamma-glutamyltransferase and early-onset coronary artery disease: a retrospective case-control study. Ann Med.

[6] Bulusu S, Sharma M (2016). What does serum γ-glutamyltransferase tell us as a cardiometabolic risk marker?. Ann Clin Biochem.

[7] Gensini GG (1983). A more meaningful scoring system for determining the severity of coronary heart disease. Am J Cardiol.

[8] Arasteh S, Moohebati M, Avan A (2018). Serum level of gamma-glutamyl transferase as a biomarker for predicting stenosis severity in patients with coronary artery disease. Indian Heart J.

[9] Taimur SD, Nasrin S, Haq MM, Rashid MA, Gomes HI, Islam F (2018). Relationship between hemoglobin A1c level and severity of coronary artery disease among hospitalized patients with acute coronary syndrome. Bangladesh Heart J.

[10] Demirkan B, Güray Y, Güray Ü, Turak O, Hajro E, Korkmaz Ş (2010). The relationship between saphenous coronary bypass graft occlusion and serum gamma-glutamyltransferase activity. Anatol J Cardiol.

[11] Koza Y, Şimşek Z, Taş MH (2013). Serum gamma-glutamyltransferase and the burden of atherosclerosis in patients with acute coronary syndrome. Turk Kardiyol Dern Ars.

[12] Huang Y, Luo J, Liu X (2018). Gamma-glutamyltransferase and risk of acute coronary syndrome in young Chinese patients: a case-control study. Dis Markers.

